# Frequency of Renal Dysfunction and its effects on outcomes after open heart surgery

**DOI:** 10.12669/pjms.37.7.3865

**Published:** 2021

**Authors:** Taimur Asif Ali, Khuzaima Tariq, Areej Salim, Saulat Fatimi

**Affiliations:** 1 Dr. Taimur Asif Ali, FCPS. Department of Cardiac Surgery, National Institute of Cardiovascular Diseases, Karachi, Pakistan; 2 Dr. Khuzaima Tariq, FCPS. Department of Cardiac Surgery, National Institute of Cardiovascular Diseases, Karachi, Pakistan; 3 Dr. Areej Salim, MBBS Agha Khan University Hospital, Karachi, Pakistan; 4 Dr. Saulat Fatimi, MD. Agha Khan University Hospital, Karachi, Pakistan

**Keywords:** Renal dysfunction, Open heart surgery, Serum creatinine, Glomerular filtration rate, Cardiopulmonary bypass

## Abstract

**Objectives:**

In this study we determined the frequency of renal dysfunction and its outcomes in terms of morbidity and mortality in patients who underwent open heart surgery at the Aga Khan University Hospital, Karachi, Pakistan.

**Methods:**

A total of 175 patients aged between 15-80 years having open heart Surgery(OHS) were included. Preoperative and postoperative serum creatinine (SCr) was noted and the glomerular filtration rate (GFR) calculated by Cockcroft-Gault equation. Their hospital course was charted and followed-up for 30-day.

**Results:**

The mean age and mean BMI were 58.1±12.6 years and 26.4±4.3 kg/m2 respectively. Females were 18.3%, out of which 51.4% hypertensive, 46.9% diabetics, 45.1% had dyslipidemia, 2.9% had preoperative renal dysfunction and 40% had moderate ejection fraction. On follow up, 30.3% developed postoperative renal dysfunction within 30-days after OHS with mean SCr and GFR as 1.6±0.7 and 56.9±24.5, respectively. In RD group more patients showed positive outcomes i.e. prolonged inotropic requirement (75.5% vs. 18%, p-value <0.005), diuretic infusion usage (47.2% vs. 3.3%, p-value <0.005), dialysis/renal replacement therapy (17% vs. 0%, p-value <0.005), requirement for prolonged ventilation (35.8% vs. 6.6%, p-value <0.005), prolonged ICU and hospital stay (15.4% vs. 1.6%, p-value <0.005 and 41.5% vs. 17.2%, p-value <0.005), sepsis (20.8% vs. 1.6%, p-value <0.005) and death (9.4% vs. 2.5%, p-value 0.05).

**Conclusion:**

Timely recognition of renal dysfunction, early renal replacement therapy, diuretics or dialysis and proper nutritional and inotropic support to maintain adequate hemostasis shows survival benefits.

## INTRODUCTION

Acute kidney injury resulting in renal dysfunction (RD) occurs in up to 30% of all patients after open heart surgery and 1% of those may require dialysis.^1^,^2^ The recent studies show incidence of post-surgery acute kidney injury varies from 5 to 42 %.^3^,^4^ Multiple causative factors may contribute to perioperative acute renal dysfunction, they may be categorized as pre-renal (reduced renal perfusion), renal (intrinsic renal insults), or post-renal (obstructive uropathy). It may occur as a result of renal ischemic injury, exotoxins (antibiotics, anesthetic agent, contrast media, and diuretics), endotoxins (myoglobin), and pre-existing renal impairment.

The institution of cardiopulmonary bypass (CPB) during open heart surgery ensues a systemic inflammatory response marked by increased membrane permeability and a transient capillary leak, it can pre-empt normal reflexes and chemoreceptor controls of the circulation by initiating coagulation cascades, circulating cell-signaling proteins, inflammatory cytokines, and entails microembolic phenomena leading to organ malperfusion. Extravasation of fluids into the interstitial space may contribute to cerebral edema, hepatic congestion, splanchnic congestion, and renal dysfunction. Either reversible or irreversible cell injury may occur. A prolonged bypass time (>4 hours) may cause hemoglobinuria, leading to impaired renal tubular function. The patients undergoing valvular or combined procedures are at two to three-fold risk of developing postoperative renal dysfunction, owing to prolonged CPB time.^5^,^6^ Previous literature has identified many risk factors associated with acute kidney injury following open heart surgery such as female gender, elevated serum creatinine and decreased GFR, perioperative hemoglobin area under the curve,^7^ use of Angiotensin-converting enzyme inhibitors,^8^ other nephrotoxic medications or intravenous contrast,^9^ LVEF < 35%, emergent surgery, shock, length of CPB and Aortic cross clamp time, co-morbidities like chronic obstructive pulmonary disease (COPD), diabetes mellitus, peripheral vascular disease, pre-existing renal insufficiency, congestive heart failure.^10^

Renal dysfunction is graded into mild, moderate or severe dysfunction measured by the level of serum creatinine (SCr) and/or estimated glomerular filtration rate (GFR).^11^ Its moderate to severe level (SCr >2.2 mg/dL or GFR <30ml/min/1.73 m^2^) or end-stage renal disease are found to be most devastating among cardiac surgery patients. There is increased risk of sepsis, prolonged ventilation and prolong ICU stay and re-admission.^12^ In the present study we intend to find out the effect of postoperative renal dysfunction on patients’ outcomes.

## METHODS

After taking permission from the competent authority and attaining an exemption from the Ethics Review Committee (ERC) (Ref# 2205-Sur-ERC-12 dated July 30, 2020), this study was undertaken at Department of Cardiothoracic Surgery of Aga Khan University Hospital, Karachi, Pakistan. Patients aged between 15-80 years with left ventricular ejection fraction (LVEF) >30% who underwent OHS utilizing cardiopulmonary bypass with preoperative SCr upto 1.5mg/dL were deemed fit for inclusion. Patients below 15 and above 80 years with preoperative SCr >1.5mg/dl or already on dialysis, candidates of emergency surgery, off-pump surgery with LVEF <30% were excluded. Duration of the study spanned over six months.

Initiated with an informed consent, the patients were recruited on the basis of eligibility criteria of preoperative serum creatinine (SCr) <1.5mg/dl measured from laboratory reports and calculation of GFR by the Cockcroft-Gault equation using age, weight and sex; [formula =(140-age) x Weight in kg x (0.85 if female) / 72 x Cr)]. Baseline patient characteristics were inquired verbally through a structured questionnaire. Strict confidentiality of the study subjects and information was maintained.

Two samples of SCr were chosen, one preoperative and second postoperative highest SCr value. If any of the post-operative SCr readings were found to be positive (i.e, SCr >1.5; GFR 30-89ml/min/1.73 m^2^) then the patient has been considered as having renal dysfunction. They were all followed for 30 days in the hospital or at clinic if discharged, and assessed for either the presence or absence of adverse outcomes i.e., death, length of hospital stay after surgery ≥8-days, length of ICU stay >6-days, ventilatory support requirement ≥24-hours, sepsis, renal therapy with diuretic infusions ≥24-hours, dialysis or continuous renal replacement therapy (CRRT) for anuric renal impairment and sepsis (seen by positive microbial growth either in blood, urine, tracheal or wound cultures). All patients have been operated by a team of consultant cardiac surgeons having post-fellowship experience of about 5-years in the relevant field.

## RESULTS

The mean age of our population was 58.1±12.6 years with mean BMI of 26.4±4.3 kg/m^2^ and 81.7% were males as shown in Table-I. The peri-operative risk factors of our study population showed 51.4% hypertensive and presence of dyslipidemia in 45.1%. Similarly, the risk factors of DM and smoking were present in 46.9% and 33.1% respectively. Moderate to severe LV dysfunction in a preoperative state also predisposes a patient to renal dysfunction. 23.4% of our study population had a moderate LV dysfunction (i.e. 30-40%).

The preoperative normal functioning of the kidneys is represented by the mean preoperative creatinine at the day of admission which was 1.1±0.2 mg/dL. It increased to a mean of 1.6±0.7 mg/dL postoperatively. Similarly, the glomerular filtration rate (GFR), as a second predictor and tool, was measured by Cockcroft-Gault equation was 78.7±29.3 ml/min/1.73m^2^ preoperatively and showed reduction postoperatively to 56.9±24.5 ml/min/1.73m^2^.

**Table I:** Baseline data and demographic characteristics.

*Characteristics*	*N=175*
Age	58.1 ± 12.6
Body mass index (BMI)	26.4 ± 4.3
***Gender***	
Female	32 (18.3%)
Male	143 (81.7%)
***Risk profile***	
Smoking	58 (33.1%)
Diabetes mellitus	82 (46.9%)
Hypertension	90 (51.4%)
Dyslipidemia	79 (45.1%)
HoRF	5 (2.9%)
***Ejection fraction (EF)***	
30-40%	41 (23.4%)
41-60%	70 (40.0%)
≥60%	62 (35.4%)
Pre-op Creatinne	1.1 ± 0.2
Pre-op GFR	78.7 ± 29.3
Post-op Creatinine	1.6 ± 0.7
Post-op GFR	56.9 ± 24.5

The outcomes within 30-days of open heart surgery is shown in Table-II. Occurrence of RD was seen in 30.3% of patients. There were more patients showing positive outcomes in RD group as compared to non-RD group i.e. Prolonged inotropic requirement (75.5% vs. 18%, p-value <0.005), dialysis / RRT requirement was found in 5.1% and the mortality was 4.6%. The requirement of either only continuous diuretic infusions or the institution of dialysis or renal replacement therapy was seen in 16.6% and 5.1% respectively. Patient to be retained either in the ICU for a period of greater than 6-days or in the hospital for >8-days marking prolonged stay was seen in 6.3% and 24.6% respectively, in the whole group. Sepsis was reported in 7.4% patients and death in 4.6% patients.

**Table II:** Post-operative 30 days outcomes.

*Outcomes*	*N=175*
Renal dysfunction	53 (30.3%)
Inotrope use (≥24 hours)	62 (35.4%)
Diuretic infusion use	29 (16.6%)
Dialysis / RRT	9 (5.1%)
Positive pressure ventilation (≥24 hr)	27 (15.4%)
***ICU stay***	
≤6 days	164 (93.7%)
≥6 days	11 (6.3%)
***Hospital stay***	
≤8 days	132 (75.4%)
≥8 days	43 (24.6%)
Sepsis	13 (7.4%)
Death	8 (4.6%)

We also analyzed the association of occurrence of RD with multiple risk factors or co-morbidities. Table-III. In those patients who had postoperative RD, greater than 50% were diabetics. The majority 71.7% had history of hypertension, 58.5% were dyslipidemics and 41.5% had history of smoking. Patients having mild to moderate LV dysfunction were seen to be more predisposed to RD as it occurred in more than 82% as compared to patients having a normal LV ejection fraction of >60%.

**Table III:** Clinical characteristics and analysis of post-operative 30 days outcomes in renal dysfunction and non-dysfunction groups.

*Characteristics*	*Renal Dysfunction*		*P-value*

	*Yes*	*No*	
Total (N)	53	122	-
***Gender***			
Male	44 (83%)	99 (81.1%)	0.769
Female	9 (17%)	23 (18.9%)	
***Risk factors***			
Diabetes	28 (52.8%)	54 (44.3%)	0.297
Hypertension	38 (71.7%)	52 (42.6%)	<0.005
Dyslipidemia	31 (58.5%)	48 (39.3%)	0.019
Smoking	22 (41.5%)	36 (29.5%)	0.121
***Ejection fraction***			
30-40%	17 (32.1%)	24 (19.7%)	<0.005
41-60%	32 (60.4%)	38 (31.1%)	
>60%	4 (7.5%)	60 (49.2%)	
Inotrope use >24-hrs	40 (75.5%)	22 (18%)	<0.005
Diuretic / Diuretic infusion use	25 (47.2%)	4 (3.3%)	<0.005
Dialysis / Renal replacement therapy	9 (17%)	0 (0%)	<0.005
Positive pressure ventilation >24-hrs	19 (35.8%)	8 (6.6%)	<0.005
***ICU stay***			
>6-days	8 (15.1%)	2 (1.6%)	0.001
<6-days	45 (84.9%)	120 (98.4%)	
***Length of hospital stay***			
>8-day	22 (41.5%)	21 (17.2%)	0.001
<8-days	31 (58.5%)	101 (82.8%)	
Sepsis	11 (20.8%)	2 (1.6%)	<0.005
Death	5 (9.4%)	3 (2.5%)	0.05

With analysis of the comorbidities and adverse outcomes, it was seen that variables of preoperative presence of hypertension, dyslipidemia and preoperative LVEF were also significant (*p<0.05*). The perioperative variables of inotropic use > 24-hrs and the requirement of positive pressure ventilation for > 24-hrs was also seen statistically significant (*p<0.05*. Due to the development of RD, the requirement of use of diuretics infusions and further requirement of dialysis or RRT was seen to be statistically significant (*p<0.05)*. All these lead to a statistically significant (*p<0.05)* prolong ICU and hospital stay as well as development of sepsis leading to death.

## DISCUSSION

This study showed that patients undergoing open heart surgical procedures at our setup are at increased risk for developing postoperative RD. There is complex interplay of number of factors which might explain its association with open heart surgery importantly preoperative health of kidneys, associated comorbidities, effects of CPB, myocardial revascularization, reperfusion injury, fluid overload, retention of uremic compounds, acidosis, electrolyte imbalance, increased risk for sepsis and anemia etc. The National Kidney Foundation in USA had formulated guideline for the optimization of patients who have preoperative dysfunction and divided it into mild, moderate or severe categories. To properly assess and compare the incidence and outcomes of Acute Kidney Injury worldwide, the Acute Dialysis Quality Initiative Group proposed a standard classification termed “RIFLE” in 2004, which stands for the acronym “Risk, Injury, Failure, Loss of function and End stage kidney disease”, and is based on two criteria: serum creatinine levels (SCr) and urine output.^13^ henceforth, the term Acute renal failure was officially replaced by Acute Kidney Injury(AKI).^14^ The currently used criteria, were published in 2012 by the Kidney Disease Improving Global Outcome (KDIGO) workgroup which contains guidelines on risk assessment, evaluation, prevention and treatment. Definition and staging of AKI are based on the Risk, Injury, Failure, Loss, End-stage renal disease(RIFLE) and Acute Kidney Injury Network (AKIN) criteria.^15^ It is interestingly noted that even a small rise in creatinine is associated with a higher mortality. The degree of rise in creatinine perioperatively closely correlates with increased mortality. A preoperative creatinine >1.5 mg/dL entails a cumulative mortality risk ranging from 5–30%.^16^ The estimated mortality risk is about 5% for patients with a creatinine of 1.5–2.5 mg/dL, and approximately 15–30% in non–dialysis-dependent patients with a creatinine >2.5 mg/dL. *Lassnigg et al*^17^ observed that in patients showing a rise in serum creatinine > 0.5mg/dL above the baseline had a 2.77-fold increase in mortality and those who had even more than a greater than 0.5mg/dL from baseline had an increased mortality of 18.64-folds in early postoperative period.

*Headley et al*^18^ studied the effects of CPB on patients undergoing different types of open heart surgery. Their series showed a mortality rate which ranged from 1.3% in simple procedures to as high as 11% for complex procedures in association with 11.3% development of renal dysfunction. *Simon et al*^19^ showed that the length of ICU and hospital stay and requirement of mechanical ventilation was very much influenced by the health of the kidneys. A relationship of developing RD by association of gender, age and BMI was also quite comparable.

The management of established postoperative renal dysfunction, so far has been supportive, including optimization of hemodynamic status, adequate hydration, correction of metabolic derangements, correction of acid-base balance, avoidance of nephrotoxic drugs and tight glycemic control. The basic treatment regime remains the same stressing upon preventive measures such as limiting nephrotoxic medication and iodinated contrast agents with optimizing volume status.^20^ recent areas of research including remote ischemic preconditioning and pharmacological interventions have showed limited efficacy in preventing renal dysfunction. The multicenter double-blind placebo-controlled clinical trial *STOP-AKI trial*^21^ has been recently published evaluating the safety and efficacy of human recombinant alkaline phosphatase as anti-inflammatory treatment for patients with septic AKI. This trial and many other studies failed to show promising results in improving kidney function. *Leu et al^22^* in his meta-analysis has suggested that perioperative administration of dexmedetomidine in adult cardiac surgery might be beneficial in reducing the incidence of AKI, opening doors to further studies.

The well-timed initiation of CRRT^23^,^24^ has been proven to be an effective modality to treat acute kidney injuries, with its use increasing over time. It has shown to decrease morbidity and improve survival.

## CONCLUSION

Postoperative renal dysfunction is a harbinger of poor prognosis after open heart surgery. The acuteness of symptoms, gravity of cardiovascular disease and associated factors related to the CPB put the kidneys at extreme risk. The frequency of renal dysfunction resulting as cause of open heart surgery is 30.3% which is quite high. It is emphasized to establish early diagnosis in order to institute corrective measures offering adequate hydration, and avoidance of potentially toxic metabolites and drugs with multi-disciplinary approach including cardiac surgeon, cardiologist, anesthetist, nephrologist, intensivist and nurses. The strategy need to first define renal dysfunction and then to design a uniform pathway to avoid morbidities to prevent development of full-fledged renal failure in the narrow time frame.

### Authors’ Contribution:

**TAA:** Designed and conceived with data collection, analysis and editing.

**KT:** Helped in statistical analysis, manuscript writing and formal lay out.

**AS:** Helped in data collection and biostatistics

**SF:** Study team supervisor, proof reading and final approval.
